# The association between drug use and mortality in a norwegian prison cohort: a prospective cohort study

**DOI:** 10.1186/s40352-023-00223-y

**Published:** 2023-04-14

**Authors:** Torill Tverborgvik, Marianne Riksheim Stavseth, Anne Bukten

**Affiliations:** 1 Norwegian Centre for Addiction Research, University of Oslo, P.O. Box 1074, Blindern, Oslo, 0316 Norway; 2 Section for Clinical Addiction Research, Oslo University Hospital, P.O. Box 4959, Nydalen, Oslo, 0424 Norway

**Keywords:** DUIT, Screening, Substance use disorders, Prison, Prison health, Mortality

## Abstract

**Background:**

Elevated mortality rates are found among people who have experienced incarceration, even long after release from prison. The mechanisms related to this excess mortality are complex products of both individual and situational factors. The aim of this study was to describe all-cause and cause-specific mortality among people with a history of imprisonment, and to examine both individual and situational factors associated with mortality.

**Methods:**

In this prospective cohort study we used baseline survey data from the Norwegian Offender Mental Health and Addiction (NorMA) study (N = 733) linked with data from the Norwegian Cause of Death Registry during eight years of follow-up (2013–2021).

**Results:**

At end of follow-up, 56 persons (8%) of the cohort were deceased; 55% (n = 31) due to external causes such as overdoses or suicides, and 29% (n = 16) to internal causes such as cancer or lung disease. Having a score > 24 on the Drug Use Disorders Identification Test (DUDIT), indicating likely drug dependence was highly associated with external causes of death (OR 3.31, 95% CI 1.34–8.16), while having a job before baseline imprisonment had a protective effect on all-cause mortality (OR 0.51, ,95% CI 0.28–0.95).

**Conclusions:**

High DUDIT score at baseline were highly associated with external causes of death, even years after the DUDIT screening was done. Screening incarcerated people using validated clinical tools, such as the DUDIT, together with initiation of appropriate treatment, may contribute to reduced mortality in this marginalized population.

**Supplementary Information:**

The online version contains supplementary material available at 10.1186/s40352-023-00223-y.

## Background

Worldwide, it is estimated that more than 11 million persons are imprisoned at any time (Fair & Walmsley, 2021), and the burden of social and health issues among people experiencing imprisonment is extensive. Higher prevalence of psychiatric and substance use disorders (SUDs) (Kinner & Rich, 2018), along with higher prevalence of somatic illness (Fazel & Baillargeon, 2011), are commonly found in this group. Lifetime prevalence of illicit drug use upon entry to prison was estimated to be in the range of 30 to 93% in 12 European countries in a literature review by van de Baan et al. (2022). In a meta-analysis of the prevalence of SUDs in newly incarcerated men and women across 10 countries, Fazel et al. (2017) found the pooled prevalence estimate to be 30% in incarcerated men and 51% in women, and thus far more prevalent than in the general population. In addition, homelessness, low socio-economic status and histories of victimization and trauma are common (Kinner & Young, 2018). High rates of multimorbidity, i.e., the presence of two or more long-term health conditions, is furthermore common among people with prison experience. In a study of 1046 persons released from prison in Queensland, Australia, multimorbidity was found in 69% of men and 85% of women Calais-Ferreira et al., 2022).

People with a history of incarceration have higher mortality rates than the general population, even years after last imprisonment. The most common cause of death after release is overdose (Brummer et al., 2018; Bukten et al., 2017), with risk peaking in the early post-release period (Binswanger et al., 2007; Bukten et al., 2017). Suicide, homicide, and accidents are frequent in the population for an extended period post-release (Binswanger et al., 2007; Spittal et al., 2019). Having a history of incarceration also elevates risks of death from internal causes, such as cardiovascular disease, cancers, infectious diseases, and liver disease (Binswanger et al., 2007; Bukten et al., 2022; Spittal et al., 2019). Homelessness, higher levels of infections, more use of alcohol, tobacco and drugs, and low socio-economic status are possible explanations for the elevated risk of death from internal causes among populations released from prison (Binswanger et al., 2007; Brinkley-Rubinstein, 2013).

In a study of the association between psychiatric disorders and mortality in people released from prison in Sweden, Chang et al. (2015) found that having a SUD-diagnosis significantly increased the rate of all-cause mortality. The association was independent of sociodemographic, criminological, and familial factors, supporting a causal effect of SUDs on mortality.

Incarceration of marginalized people can thus both produce and reinforce social and health inequalities, including disparities in mortality. The causal mechanisms related to excess mortality are complex and a product of individual factors, such as health status and background, combined with situational factors including penitentiary conditions, security level and experience of isolation (Brinkley-Rubinstein et al., 2019; Flam-Ross et al., 2022) highlights the importance of political and societal factors on opioid-involved overdoses among previously incarcerated people in the U.S.

Few studies have integrated both individual and situational factors when investigating mortality among people with a history of imprisonment. In this prospective cohort study, we used questionnaire data from the Norwegian Offender Mental Health and Addiction (NorMA) study combined with registry data from the Norwegian Cause of Death Registry (NCoDR). With extensive questionnaire data, including several validated screening tools, along with detailed cause-of-death data we aimed to (1) describe drug use patterns and individual factors in a cohort of people with a history of imprisonment, (2) describe all-cause and cause specific mortality in this cohort, and (3) explore individual and situational factors associated with mortality.

## Methods

The present work is a prospective cohort study based on data from the NorMA study (Bukten et al., 2015; Lokdam et al., 2021) linked with data from the Norwegian Prison Registry and the NCoDR on an individual level using the Norwegian 11-digit personal identification number (PIN).

### Settings

Norway is a Nordic country with a low imprisonment rate. As of May 2022, 3,124 individuals were imprisoned, corresponding to an imprisonment rate of 58 per 100,000 of the national population, as compared to 131 per 100,000 in the United Kingdom and 629 per 100,000 in the USA in 2021 (Fair & Walmsley, 2021). The imprisonment rate of the other Nordic countries was 72 in Denmark, 73 in Sweden and 50 in Finland (Fair & Walmsley, 2021). Women constitute a minority in Norwegian prisons, making up an annual proportion of just over 5%. All Norwegian prisons are publicly funded, and universal health care is available for all convicted persons. The prisons are rehabilitation-oriented, adopting the principle of normality and facilitating an everyday life which mirrors - as close as is feasible - life outside of prison, and with the goal of eliminating recidivism (The Norwegian Correctional Service, 2022).

### Cohort

The NorMA study included 1,495 individuals incarcerated in Norwegian prisons responding to a 116-item survey in the period 1 June 2013 to 31 July 2014 (denoted “baseline”). The survey included self-reported information on demographics, pre-baseline and baseline drug and alcohol use, as well as several validated clinical screening tools. All persons imprisoned in Norway at the time of data collection were eligible to participate, and the questionnaire was available in English, Russian, French, and German in addition to Norwegian. There were 62 prison units in Norway at the time of data collection, and 56 of these were visited by the researchers (Bukten et al., 2015).

Of the participants in the NorMA study, 733 consented to follow-up by providing a valid Norwegian PIN, thus constituting the NorMA cohort. For a more thorough description of the study design, see Bukten et al. (2015). For a description of the full NorMA study cohort, see Bukten et al. (2020). A prior study on external validity of the NorMA study cohort found it to be largely representative of the general prison population in possession of a Norwegian PIN, but not to those without (Lokdam et al., 2021).

### Data sources

The Norwegian Cause of Death Register (NCoDR), maintained by the Norwegian Institute of Public Health, constitutes death certificates reported by medical doctors after examination of the deceased. Along with date and place of death, the registry includes information on causes of death coded according to the International Classification of Diseases, 10th revision (ICD-10) (World Health Organization, 1992). Clinicians may register multiple ICD 10 codes indicating causes of death in the NCoDR, with Underlying Cause of Death being the disease or injury initiating the train of events leading directly to death. The NCoDR comprises all Norwegian residents and includes medical information on more than 98% of all deaths (Pedersen & Ellingsen, 2015).

The Norwegian Prison Registry constitutes information on all incarcerations in Norwegian prisons from 1992 onwards, with information on date of incarceration, transitions, and release along with information on convictions, prison unit and security level (Bukten et al., 2015).

### Measures

Underlying cause of death was considered for this study. For deaths where underlying cause of death was drug related additional ICD10 codes indicating type of substance involved were obtained. Combined causes were not considered. Causes of death were categorized into two main groups based on ICD-10 codes; “internal” constitutes underlying cause of death in Chaps. 1–17, while “external” constitutes underlying cause of death in Chap. 20. We chose to include drug-related deaths in the “external” group as described in Table 1. Nine deaths were categorized as “unknown” due to missing information about the cause of death. To ease the interpretation of our results, those deceased due to internal causes is denominated the internal-cause group, while those deceased due to external causes is denominated the external-cause group.

**Table 1:** Description of survey and registry variables* included in the present study

Variable	Description	Missing, % of total
Data from the NorMA Survey N = 733		
*Demographics*		
Sex	Male vs. female	
Age	Age when answering the NorMA survey	
Norwegian born	Born in Norway vs. born in other countries	2.5
Family with drug or mental health problems	Growing up in a family with drug use and/or psychiatric disorders vs. no such problems	3.5
Education	Primary school (10 years) or less vs. more than primary school	1.2
In job or education	Working or in an educational program prior to baseline imprisonment vs. not	2.2
Married	Married/cohabitant vs. not	1.1
Benefits as main income	Benefits (pensions, sickness benefits or unemployment benefits) as main income vs. other income	1.8
Homeowner	Owner of private housing vs. not	1.2
*Mental Health*		
HSCL-10	Hopkins symptoms check list, measuring symptoms of psychological distress. Cut-off (> 1.85) indicates psychological distress (Derogatis et al., 1974). Mean-imputation is used if missing on two or less of the 10 questions.	14.1
*Substance use*		
Used alcohol	Ever used alcohol, denoted “lifetime”	1.0
Age at onset, alcohol use	Age at first use of alcohol	
Used illegal drugs	Ever used illegal drugs, denoted “lifetime”	2.9
Age at onset, drug use	Age at first use of illegal drugs	
Daily use	Substance use (not including alcohol) 4 times per week or more during the 6 months leading up to baseline imprisonment vs. less than 4 times per week	
OAT	In opioid agonist treatment at time of survey	6.1
Attended treatment	Ever attended drug and/or alcohol treatment	
AUDIT	Alcohol Use Disorders Identification Test – a 10 items screening tool for identification of problematic alcohol use during the year leading up to baseline imprisonment. Cut-off (> 19) indicating dependence and need for treatment	1.9
DUDIT	Drug Use Disorders Identification Test – an 11 items screening tool for identification of problematic use of illegal drugs or prescribed medication during the year leading up to baseline imprisonment. Cut-off (> 24) indicating dependence and need for treatment	3.4
Prescription medicine	Used prescribed medication for sleeping disorders, ADHD, anxiety, depression, OAT or pain during the 6 months leading up to baseline imprisonment and/or during baseline imprisonment vs. not	
**Data from the Norwegian Death Registry (NCoDR) N = 56**		
Time of death	Date as stated in the NCoDR	
Age	Age at death. Missing for eight deaths in the Death registry, and thus calculated from age at baseline and date of death.	
Underlying cause of death	ICD10 code describing the disease or injury that initiated the train of events leading directly to death. Dichotomized into “Internal death” and “External death”.	14.3
Internal cause of death	Underlying cause of death with ICD10 codes in Chaps. 1–17 (A00-Q99), excluding codes related to drug-related deaths as stated below	
External cause of death	Underlying cause of death with ICD10 codes in Chap. 20 (V00-Y98), plus drug-related deaths as stated below	
Drug-related death	Underlying cause of death with ICD10 codes F (11–12,14–16,19), X (41^1^,42^2^,44^2^, 61^1^,62^2^, 64^2^) and Y (11^1^,12^2^). Included in “External cause of death”.	
**Data from the Norwegian Prison Registry, on baseline imprisonment N = 733**		
Security level	Released from high security unit at end of baseline imprisonment	7.6
Duration	Length of baseline imprisonment (in months) from date of imprisonment to date of release or end of follow-up.	

*Variables listed from the NorMA survey, the NCoDR (for the deceased) and the Norwegian Prison Registry

^1^In combination with contributing cause T43.6

^2^In combination with contributing cause T40.0-9

Data on baseline incarceration, i.e., the imprisonment that the participants underwent when completing the NorMA survey, including security level and duration, was obtained from the Norwegian Prison Registry. Duration of baseline incarceration was calculated as the time between date of imprisonment and date of release or end of follow-up.

The Alcohol Use Disorders Identification Test (AUDIT) and the Drug Use Disorder Identification Test (DUDIT) are validated instruments for clinical use included in the NorMA survey. Scoring over 19 points on AUDIT indicates likely alcohol dependence (Babor et al., 2001), while scoring more than 24 points on DUDIT indicates likely drug dependence (Berman et al., 2007). Participants were asked about their use of alcohol and drugs in the year leading up to their baseline imprisonment. Variables from the NorMA survey and registry data used in this study are outlined in Table 1.

### Analysis

All analyses were performed using SPSS, version 26. A number of variables were included in the descriptive analysis to give an extensive description of the deceased. We made group comparisons using Student’s t-test or Mann-Whitney U-test for continuous data and X^2^-test for categorical data. Based on these comparisons, potential covariates were chosen for the regression models. Missing data were treated using complete case analysis and are reported per variable in Table 1.

We examined the association between mortality and potential risk factors using logistic regression. Participants deceased due to internal causes of death differed significantly from those deceased due to external causes. Therefore, separate logistic regression models were fitted based on cause of death. The three regression models were defined as (1) all-cause mortality (n = 56) (2) death due to internal causes (n = 16), and (3) death due to external causes (n = 31). To avoid competing risks, unknown and external cause deaths were excluded from analysis in the second regression model, and unknown and internal cause deaths were excluded from analysis in the third model.

Potential risk factors were identified from previous literature and from between-group comparisons. However, due to the low number of mortalities, a limited number of risk factors could be included in the regression models. All potential covariates were checked for multi-collinearity, and highly correlated items were not included in the regression models. Due to the low number of deceased women (n = 2), sex was not included as a covariate.

Univariate regression models were fitted, and statistically significant covariates (p-value < 0.05) were included in the multivariable analysis. Age is known to be strongly correlated with mortality and age at baseline was therefore included in all multivariable analyses.

### Ethics

The NorMA study was approved by the Norwegian Committee of Research Ethics (REK 2012/297), by the Ministry of Justice and Public Security, and by the Directorate of the Norwegian Correctional Services. Participation in the study was voluntary and based on written informed consent. Participants were informed that their answers would not be shared with prison staff.

## Results

The NorMA cohort consisted of 733 individuals, out of which 682 (93%) were male and 602 (82%) were Norwegian born (Table 2). At end of follow-up 56 (8%) were deceased (Table 2). The deceased were more often born in Norway (96% vs. 81%) and in general older than the non-deceased at baseline (mean age 44 vs. 35 years). Fewer had a job or had been in an educational program prior to baseline imprisonment (23% vs. 42%), more had welfare benefits as main income (64% vs. 46%) and they generally had shorter baseline incarcerations (mean duration 8 vs. 11 months; Table 2).

**Table 2:** Baseline characteristics for the total cohort (N = 733), for the deceased (n = 56) and not-deceased (n = 677) separately

	Deceased (n = 56)	Not-deceased (n = 677)	Total (N = 733)
	n (%)	n (%)	n (%)
**Demographics** ^**1**^			
Male	54 (96.4)	628 (92.8)	682 (93.0)
Norwegian born	54 (96.4)	548 (80.9)	602 (82.1)*
Age at baseline. mean (SD)	43.64 (12.75)	34.85 (11.24)	35.52 (11.59)***
*< 34 years*	*13 (23.2)*	*384 (56.7)*	*397 (54.2)*
*35–44 years*	*18 (32.1)*	*157 (23.2)*	*175 (23.9)*
*45–54 years*	*14 (25.0)*	*97 (14.3)*	*111 (15.1)*
*> 55 years*	*11 (19.6)*	*39 (5.8)*	*50 (6.8)*
Family with drug or mental health problems	23 (41.1)	242 (35.7)	265 (36.2)
< 10 years of education	24 (42.9)	276 (29.1)	300 (40.9)
Married	11 (19.6)	197 (29.1)	208 (28.3)
In job or education	13 (23.2)	285 (42.1)	298 (40.7)**
Benefits as main income	36 (64.3)	316 (46.1)	352 (48.0)*
Homeowner	13 (23.2)	138 (20.4)	151 (20.6)
**Mental health** ^**1**^			
Psychological distress (HSCL10), mean (SD)	1.99 (0.82)	1.97 (0.81)	1.97 (0.81)
*Over cut-off*	*22 (39.3)*	*275 (40.6)*	*297 (40.5)*
**Baseline imprisonment** ^**2**^			
Duration in months, median (range)	8.0 (0.5–66.1)	11.2 (0.2–167.0)	11.1 (0.2-167.5)*
High level of security	26 (46.4)	245 (36.2)	271 (37.0)

^1^Data from baseline survey

^2^Data from the Prison registry

*p < .05 **p < .01 ***p < .001

The majority of deaths were due to external causes (n = 31, 55%; Table 3), with drug-related deaths being the most common cause (n = 17, 30%). Of the 17 drug-related deaths, 82% (n = 14) were overdoses from opioids. Five (16%) out of the 31 external deaths were due to suicide. Sixteen (29%) individuals died of internal causes. Of these, eight died from circulatory or respiratory disease and eight from other diseases. Mean age at death was lower for those dying from external causes (40 years vs. 57 years; Table 3).

**Table 3:** Underlying cause of death (n = 56) with mean age at death

Underlying cause of death	n (%)	Mean age at death (SD)
Total	56 (100)	47.12 (12.89)
Circulatory or respiratory disease	8 (14.3)	57.50 (12.22)
Other disease	8 (14.3)	56.00 (4.47)
**Total Internal cause of death**	**16 (28.6)**	**56.75 (8.93)**
Drug-related	17 (30.4)	38.82 (7.14)
*Opioids* ^*1*^	*14 (82.4)* ^*1*^	*38.71 (7.48)* ^*1*^
Other external cause	14 (25.0)	41.50 (13.77)
*Suicide* ^*2*^	*5 (35.7)* ^*2*^	*36.40 (11.72)* ^*2*^
**Total External cause of death**	**31 (55.4)**	**40.03 (10.54)**
Missing/Unknown cause of death	9 (16.1)	54.44 (11.66)

^1^Subgroup of “Drug-related deaths”

^2^Subgroup of “Other external cause”

Table 4 displays the self-reported history of drug and alcohol use prior to baseline imprisonment for the deceased. Most had used alcohol in their lifetime. Among persons dying from external causes, 94% had a history of drug use, compared to 50% of those dying from an internal cause (Table 4). Thirty one percent (n = 5) in the internal-cause group had ever attended alcohol or drug treatment, as had 61% (n = 19) in the external-cause group.

**Table 4:** History of drug and alcohol use for the deceased in the cohort

	Total (N = 56)*	Internal (n = 16)	External (n = 31)	p-value
**Ever used alcohol**	53 (94.6)	15 (93.8)	30 (96.8)	0.570
**Age at first alcohol use, mean (SD)**	13.20 (2.95)	14.07 (3.79)	13.00 (2.22)	0.087
**Ever used drugs**	43 (76.8)	8 (50.0)	29 (93.5)	0.001***
**Age at first drug use, mean (SD)**	16.13 (6.27)	16.83 (3.87)	16.00 (6.54)	0.767
**Daily drug use 6 months prior to baseline**	28 (50.0)	6 (37.5)	20 (64.5)	0.073
Opioids	10 (17.9)	0 (0.0)	10 (32.3)	0.009**
Other^1^	18 (32.1)	6 (37.5)	10 (32.3)	0.073
**In OAT at time of baseline**	10 (17.9)	4 (25.0)	8 (19.4)	0.346
**Attended alcohol and/or drug treatment**	27 (48.2)	5 (31.3)	19 (61.3)	0.049*
**Prescribed medication** ^**2**^	37 (66.1)	11 (68.8)	20 (64.5)	0.518
**Likely dependence of alcohol and/or drugs** ^**3**^	34 (60.7)	7 (43.8)	24 (77.4)	0.024*
AUDIT > 19 points	11 (19.6)	3 (18.8)	7 (22.6)	0.538
DUDIT > 24 points	29 (51.8)	4 (25.0)	23 (74.2)	0.002**
**Duration of baseline imprisonment, median months (range)**	8.0 (0.5–66.1)	7.9 (1.0-47.3)	6.3 (0.5–49.4)	0.873
**High level of security at release from baseline imprisonment**	26 (46.4)	8 (50.0)	15 (48.4)	0.818

*All deaths included in the total-calculations (N = 56)

^1^ Stimulants, Benzodiazepines, Cannabis, and other drugs not on prescription

^2^ For sleeping disorders, ADHD, anxiety, depression, OMT or pain

^3^ Score of > 19 points on AUDIT and/or score of > 24 points on DUDIT at baseline

*p < .05 **p < .01 ***p < .001

There were no differences among the groups in proportions of individuals scoring likely dependent on the AUDIT (Table 4). However, a significantly higher proportion among those deceased from external causes scored likely dependent on the DUDIT (74% vs. 25%). Twenty-eight (50%) of the deceased had reported daily drug use prior to baseline; 10 had used opioids daily, and 16 reported daily use of other drugs (Table 4). All 10 reporting “daily opioid use” died from external causes, and seven of which were overdoses with opioids (Supplementary Table 1). Among those that had reported daily use of drugs other than opioids, six (38%) died from internal causes, while ten (62%) died from external causes, including five deaths from overdoses with opioids (Supplementary Table 1).

The adjusted logistic regression model for all-cause mortality (n = 56) showed that having a job or being in an educational program prior to baseline imprisonment had a protective effect (OR 0.51, 95% CI 0.28–0.95; Table 5), while older age was associated with a higher risk of all-cause mortality (OR 1.07, 95% CI 1.04–1.09). Duration of baseline imprisonment had a protective effect on all-cause mortality (OR 0.98, 95% CL 0.96–0.99).

**Table 5:** Separate Logistic regression models with odds ratios (ORs) and 95% confidence intervals (CIs).

	Cause of death									
	All-cause (n = 56)				Internal (n = 16)^a^		External (n = 31)^b^			
	Crude OR (95% CI)	p-value	Adjusted OR^4^ (95% CI)	p-value	Crude OR (95% CI)	p-value	Crude OR (95% CI)	p-value	Adjusted OR^4^ (95% CI)	p-value
**Age at baseline**	**1.06 (1.04–1.08)**	< 0.001***	**1.07 (1.04–1.09)**	< 0.001***	**1.12 (1.07–1.16)** ^**5**^	< 0.001***	1.02 (0.99–1.05)	0.327	1.03 (0.99–1.07)	0.115
**In job or education** ^**1**^	**0.51 (0.28–0.93)**	0.027*	**0.51 (0.28–0.95)**	0.033*	0.76 (0.27–2.12)	0.605	**0.31 (0.12–0.75)**	0.010**	0.52 (0.20–1.35)	0.177
**AUDIT likely dependence** ^**2**^	0.98 (0.50–1.95)	0.962	-	-	0.93 (0.26–3.31)	0.909	1.17 (0.50–2.78)	0.717	-	-
**DUDIT likely dependence** ^**2**^	1.47 (0.84–2.56)	0.179	-	-	0.42 (0.13–1.32)	0.138	**3.63 (1.60–8.24)**	0.002**	**3.31 (1.34–8.16)**	0.009***
**Duration** ^**3**^	**0.98 (0.97-1.00)**	0.027*	**0.98 (0.96–0.99)**	**0.008**	0.98 (0.95–1.01)	0.174	0.98 (0.96-1.00)	0.076	-	-

^a^ Deaths from external causes are excluded in the model

^b^ Deaths from internal causes are excluded in the model

^1^ Prior to baseline imprisonment

^2^Alcohol Use Disorders Identification Test (AUDIT) and Drug Use Disorders Identification Test (DUDIT) measured at baseline

^3^ Duration (months) of baseline imprisonment

^4^ Adjusted for age and covariates significant in crude analysis

^5^ Only age at baseline was significantly associated with death from internal causes and an adjusted model was thus not run for this group

*p < .05 **p < .01 ***p < .001

Age at baseline was the only variable significantly associated with death in the internal-cause model (OR 1.12, 95% CI 1.07–1.16).

In the adjusted external-cause model, a high DUDIT score was associated with a higher risk of death (OR 3.31, 95% CI 1.34–8.16: Table 5). Having a job before baseline showed a protective effect on external-cause death only in the unadjusted model (OR 0.31, 95% CI 0.12–0.75).

## Discussion

This prospective cohort study aimed at describing mortality in a Norwegian prison cohort. By combining survey data with register data on an individual level, we found that a DUDIT score indicating likely dependence (score of > 24) was significantly associated with a highly elevated risk of external cause mortality up to eight years after the initial DUDIT screening. We furthermore found that being in a job before baseline imprisonment was protective of all-cause mortality.

### Drug use and mortality in prison

Elevated risk of death from suicide is found for people in prison, spiking in the first few days of incarceration (Bukten & Stavseth, 2021), with withdrawal from drug use been identified as a possible trigger (Larney et al., 2015). Mortality rates are also high after release from prison, particularly for people with SUDs. In the NorMA cohort, high rates of death from both suicide and drug-related causes were found, in line with previas literature. In the cohort, more than 90% of those who died from external causes had a history of drug use, and one-third reported daily use of opioids prior to incarceration. Within drug-related deaths, more than eight in 10 were overdoses from opioids. Norway has a high number of drug-related deaths each year (Amundsen, 2022), and in a toxicological study of all overdose deaths in Norway during the years 2000–2019, Edvardsen and Clausen (2022) found opioids in 93% of the cases.

We have not differentiated between death inside and outside of prison in the present study. It might be that specific causes of death differ inside and outside of prison, but because we collapsed all causes of death into the broad internal-cause and external-cause groups, small differences in specific causes would not affect our results.

### DUDIT and screening

After adjusting for relevant covariates, we found that people who had scored > 24 on the DUDIT at baseline, indicating likely dependence, had a three-times higher risk of external death, even up to eight years after the DUDIT screen. People with SUDs in prison tend to have more wide-ranging mental and social problems, including lower educational qualifications, lower rates of employment, more housing difficulties, poorer physical health, and more behavioral, psychological, and psychiatric problems, compared to other inmates (Kinner & Rich, 2018). Time in prison can be regarded as a window of treatment opportunity, especially for those that in the community can be hard to reach (Carpentier et al., 2018). Treatment inside prison can thus have positive outcomes on the individual level and for the community in which the person will be released (Binswanger et al., 2016; Bukten et al., 2012; Chang et al., 2015). Systematic screening in prison could be used as an important first step to identify those in need of treatment and implement the actions necessary for avoiding negative outcomes (Carpentier et al., 2018). However, according to the WHO validated screening tools for drug or alcohol problems are rarely used in prisons (World Health Organization, 2019). The DUDIT is a validated and frequently recommended instrument for screening for SUDs, but the length of the instrument may make it unsuitable in some contexts. Shorter versions have been tested, and a recent study by Pape et al. (2022) found that a five-item version of the DUDIT (items 1–4 (DUDIT-C) + item 5) identified 97% of all cases of likely dependence.

### Attachment to the labor market

Having a job or being in an educational program prior to baseline incarceration showed a protective effect on all-cause and external causes of death, even after adjusting for other covariates. In a study of social determinants of drug-related mortality in the Finnish population, Rönkä et al. (2017) found unemployment to be strongly associated with drug-related deaths, and Aram et al. (2020) found higher risk of overdose mortality among American adults with weak labor market attachment, after adjusting for several characteristics, including both education and poverty level. Employment have furthermore been found to positively affect endurance in opioid agonist treatment (Eastwood et al., 2018). The positive effects of employment may result from increased structure throughout the day, having more stable economy, and engaging in a positive social network, as well as providing a sense of dignity, belonging, and meaning. Berg and Huebner (2011) found strong social capital in the form of family bond, to be a predictor of employment and lower recidivism.

### Age

We found that the mean age at death was lower for those dying from external causes compared to internal causes (40 years vs. 57 years). These finding are consistent with a larger study based on data from the total Norwegian prison population (2000–2016) which reported that the mean age of death from external causes was 39 years, and 55 years from internal causes (Bukten et al., 2022). In a large life-expectancy study of people dependent on opioids in Australia, Lewer et al. (2020) found life expectancy at age 18 to have a deficit of 14.7 years for men and 15.8 years for women when compared to the general population, and drug-related deaths contributed approximately one third of potential years of life lost. Several studies have found people experiencing incarceration to be biologically older than expected given their numerical age (Berg et al., 2021). It is suggested that incarceration triggers a stress response leading to physiological deterioration, and thus to accelerated aging (Berg et al., 2021; Massoglia & Remster, 2019). Controlling for time spent on parole and a range of demographic and criminal justice-related factors, Patterson (2013) found that the expected life span of a person with prison experience decreased with approximately two years for every year spent in prison, but that the expected life span returned to pre-prison levels after a certain period of time.

### Strengths and limitations

This study has a prospective cohort design; a study design considered the gold standard among observational studies (Hammoudeh et al., 2018). The design minimizes recall bias and has the advantage of collecting data on the exposure prior to the outcome. A potential disadvantage is loss to follow up. However, with the outcome being collected from a national, mandatory registry, loss to follow-up is rare in this study. The NorMA cohort has furthermore been assessed and found to be largely representative of the general prison population in possession of a Norwegian PIN, but not to those without (Lokdam et al., 2021). Our results can therefore be generalized to the Norwegian prison population holding a PIN.

Another strength of the present study was the possibility of using both survey and national registry data to investigate risk factors associated with mortality (Lund & Bukten, 2015). The sole use of register data often limits the availability of rich demographic and sociocultural variables. The NorMA study included a wide range of variables, including validated clinical screening tools such as AUDIT and DUDIT to assess alcohol and drug use. Because of this, we were able to obtain information that would be impossible to identify in nationwide registers alone.

All deaths in NCoDR are classified according to the most recent ICD criteria, and death categories are reported according to individual ICD codes, minimizing the risk of information bias (Kinner et al., 2013).

However, when interpreting our results some limitations should be considered. First, using self-report data on drug use before imprisonment may limit validity and reliability. Respondents may have had difficulty recalling information about pre-prison drug use, which may lead to some recall bias and underestimations. Secondly, our study is vulnerable to low statistical power. Death is a rare event and during the eight-year follow-up only 56 deaths were recoded. In addition, cause of death was missing for 15% of the deceased, mostly due to a time lag in the update of the NCoDR. To address this gap, we minimized the number of covariates in the regression analysis. Thirdly, we did not differentiate between time in or out of prison during follow-up. Although mortality rates for people who have been experiencing incarceration are higher than for the general population, being incarcerated may have a protective effect on mortality for incarcerated populations (Binswanger et al., 2011; Kinner et al., 2013).

Finally, we were not able to differentiate between men and women in the present study due to the low number of deceased women. Studies have suggested that the age distribution of drug-related deaths may differ by sex, and younger age has been found to be an important determinant of mortality among incarcerated women in Australia (Kariminia et al., 2007).

## Implications

In Norway, people in prison are entitled to adequate health care. Thus, time in prison could be seen as a window of opportunity for detecting and treating SUDs. A recent report from the EMCDDA emphasized that understanding the prevalence and patterns of drug use among people in prison is a key policy requirement, not just for ensuring adequate care inside prison, but to help the transition from prison back into society (EMCDDA, 2021). Our results support this policy and additionally show that such knowledge is also important for preventing mortality.

Screening for alcohol and drug problems is done to some extent in Norwegian prisons, but not with validated clinical tools. As shown by Pape et al. (2021) and Pape et al. (2022), short versions of both AUDIT and DUDIT are available and highly valid in a prison context. Our finding of the strong association between a high DUDIT score and death by external causes such as overdose and suicide, justifies the need for screening for SUDs during incarceration and for the availability of evidence-based treatment for those in need. In addition, systematic validated screening could reveal a more accurate prevalence of SUDs in prison populations and thus provide stakeholders with information needed to secure the availability of necessary treatment.

Our research found a strong protective effect of employment prior to imprisonment. This effect highlights the importance of supporting labor market attachment upon release from prison. We recommend that post-release programs focus on strengthening connection to the labor market and the acquisition of relevant competence.

Even with Norway’s low prison rates, humane criminal legal policies and universal health service, mortality in the prison population is comparable to other countries, most likely due to selective processes (Bukten et al., 2022). Results from this study might therefore be generalized across countries, despite different prison rates and prison structures.

## Electronic supplementary material

Below is the link to the electronic supplementary material.

Supplementary Material 1

## Data Availability

The data for the present study is not available for sharing due to ethical considerations.
