# Supplementary material for: The association between drug use and mortality in a norwegian prison cohort: a prospective cohort study
Source: Health Justice. 2023 Apr 14;11:22. doi: 10.1186/s40352-023-00223-y (PMC10103423; doi:10.1186/s40352-023-00223-y)
Supplement: Supplementary file 1 — Supplementary Material 1 [file 40352_2023_223_MOESM1_ESM.docx]

Supplementary material

Supplementary Table 1: Underlying cause of death for those stating daily drug use prior to baseline imprisonment (n=28)

| **Cause of death** (ICD10) | **Daily use of opioids (%)** | **Daily use of other drugs (%)** |
| --- | --- | --- |
| **Internal causes** | **0 (0.0)** | **6 (31.3)** |
| **Drug-related** | **8 (80.0)** | **7 (50.0)** |
| *Opioids^1^* | *7 (70.0)* | *5 (31.3)* |
| **Other external causes** | **2 (20.0)** | **3 (18.8)** |
| **Total number of deceased** | **10 (100)** | **16 (100)** |

^1^Subgroup of “Drug-related deaths”
